# Association of Public Reporting of Medicare Dialysis Facility Quality Ratings With Access to Kidney Transplantation

**DOI:** 10.1001/jamanetworkopen.2021.26719

**Published:** 2021-09-24

**Authors:** Joel T. Adler, Lingwei Xiang, Joel S. Weissman, James R. Rodrigue, Rachel E. Patzer, Sushrut S. Waikar, Thomas C. Tsai

**Affiliations:** 1 Department of Surgery, Brigham and Women’s Hospital, Boston, Massachusetts; 2 Center for Surgery and Public Health, Brigham and Women’s Hospital, Boston, Massachusetts; 3 Department of Surgery, Beth Israel Deaconess Medical Center, Boston, Massachusetts; 4 Department of Surgery, Emory Medical School, Atlanta, Georgia; 5 Department of Medicine, Emory Medical School, Atlanta, Georgia; 6 Department of Medicine, Boston Medical Center, Boston, Massachusetts; 7 Department of Health Policy and Management, Harvard T. H. Chan School of Public Health, Boston, Massachusetts

## Abstract

**Importance:**

Improving the quality of dialysis care and access to kidney transplantation for patients with end-stage kidney disease is a national clinical and policy priority. The role of dialysis facility quality in increasing access to kidney transplantation is not known.

**Objective:**

To determine whether patient, facility, and kidney transplant waitlisting characteristics are associated with variations in dialysis center quality.

**Design, Setting, and Participants:**

This population-based cohort study is an analysis of US Renal Data System data and Medicare Dialysis Facility Compare (DFC) data from 2013 to 2018. Participants included all adult (aged ≥18 years) patients in the US Renal Data System beginning long-term dialysis in the US from 2013 to 2017 with follow-up through the end of 2018. Patients with a prior kidney transplant and matched Medicare DFC star ratings to each annual cohort of recipients were excluded. Patients at facilities without a star rating in that year were also excluded. Data analysis was performed from January to April 2021.

**Exposures:**

Dialysis center quality, as defined by Medicare DFC star ratings.

**Main Outcomes and Measures:**

The primary outcome was the proportion of patients undergoing incident dialysis who were waitlisted within 1 year of dialysis initiation. Secondary outcomes were patient and facility characteristics.

**Results:**

Of 507 581 patients beginning long-term dialysis in the US from 2013 to 2017, 291 802 (57.4%) were male, 266 517 (52.5%) were White, and the median (interquartile range) age was 65 (55-75) years. Of 5869 dialysis facilities in 2017, 132 (2.2%) were 1-star, 436 (7.4%) were 2-star, 2047 (34.9%) were 3-star, 1660 (28.3%) were 4-star, and 1594 (27.2%) were 5-star. Higher-quality dialysis facilities were associated with 47% higher odds of transplant waitlisting (odds ratio [OR], 1.47; 95% CI, 1.39-1.57 for 5-star facilities vs 1-star facilities; *P* < .001). Black patients were less likely than White patients to be waitlisted for transplantation (OR, 0.74; 95% CI, 0.72-0.76). In addition, patients at for-profit (OR, 0.78; 95% CI, 0.74-0.81) and rural (OR, 0.63; 95%, CI 0.58-0.68) facilities were less likely to be waitlisted for transplantation compared with those at nonprofit and urban facilities, respectively.

**Conclusions and Relevance:**

In this cohort study, patients at higher-quality dialysis facilities had higher odds than patients at lower-quality facilities of being waitlisted for kidney transplantation within 1 year. Waitlisting rates for kidney transplantation should be considered for integration into the current Centers for Medicare & Medicaid Services DFC star ratings to incentivize dialysis facility referral to transplant centers, inform patient choice, and drive quality improvement by increasing transplant waitlisting rates.

## Key Points

**Question:**

Are patient, facility, and/or kidney transplant waitlisting characteristics associated with variations in dialysis center quality?

**Findings:**

In this cohort study of US Renal Data System and Medicare Dialysis Facility Compare data, higher-quality dialysis facilities were associated with approximately 47% higher odds of transplant waitlisting.

**Meaning:**

These findings suggest that waitlisting rates for kidney transplantation should be considered for integration into the current Centers for Medicare & Medicaid Services Dialysis Facility Compare star ratings to improve care quality and access.

## Teaser

This cohort study examines whether patient, facility, and kidney transplant waitlisting characteristics are associated with variations in dialysis center quality.

## Introduction

Improving the quality of dialysis care and access to transplantation for patients living with end-stage kidney disease (ESKD) has been a major US health policy goal. A key component is through public reporting of quality metrics and a pay-for-performance program to incentivize increased transplant waitlisting rates: the Expanding Treatment Choices (ETC) model under the Advancing American Kidney Health initiative will adjust Medicare dialysis payments according to dialysis facility–level transplant waitlisting measured by the percentage of prevalent patients waitlisted (PPPW). Prior studies^1,2^ focused on the quality of care and accessibility of high-quality dialysis facilities, but it is unknown how dialysis facility quality is associated with waitlisting for kidney transplantation. Understanding this association would offer not only a patient-centered metric of quality and access across the care continuum for patients with ESKD, but also would assess alignment of incentives for dialysis facility quality improvement and pay-for-performance programs to increase access to transplantation.

Dialysis facility quality has been assessed and reported by the Centers for Medicare & Medicaid Services since 2000. The Medicare Dialysis Facility Compare (DFC) star ratings, which are based on clinical metrics of dialysis adequacy and patient outcomes, are meant to inform patient choice about dialysis facilities.^3,4^ Although the ETC model will shift payment of dialysis facilities to a pay-for-performance model based on transplant waitlisting rates, it is unknown how the quality metrics guiding patient choice of dialysis facilities relate to the downstream facility-level metric of transplant waitlisting. Empirical data would be helpful to assess whether higher-quality dialysis facilities, which already achieve better dialysis clinical outcomes,^3,4^ are also associated with higher transplant waitlisting rates.

As a critical site of care for patients with ESKD, dialysis centers serve as important gatekeepers for referrals to kidney transplant centers. Because private acquisition of transplant centers is associated with decreased transplant waitlisting,^5^ there is concern that perverse financial incentives will hinder access to transplantation. High-quality dialysis centers may be better at waitlisting patients with ESKD because of a positive spillover of better clinical attention to quality of care. Preliminary single-state data found an association of higher-quality dialysis centers with increased referral for kidney transplant evaluation,^6^ but national data have been lacking. Therefore, we sought to provide empirical data to inform the development of Centers for Medicare & Medicaid Services quality measures and ESKD pay-for-performance by asking 3 questions. First, what are the patient and facility characteristics associated with variations in dialysis center quality? Second, do 1-year kidney transplant waitlisting rates vary across dialysis centers? Finally, are higher-quality dialysis centers more likely than lower-quality centers to waitlist patients for transplantation?

## Methods

### Data Sources

The data reported here have been supplied by the US Renal Data System (USRDS). The USRDS contains information on every patient with ESKD the US. All patients with ESKD, regardless of insurance coverage and age, are included within the USRDS. Race and ethnicity were determined by either patient self-report or their dialysis provider on the Centers for Medicare & Medicaid Services 2728 form at dialysis initiation. Race and ethnicity were assessed in this study because of their associations with disparities in access to kidney transplantation and because these disparities reflect deeper issues with access to care. The institutional review board of MassGeneral Brigham approved the limited use data set; because this was a secondary analysis of deidentified data, no informed consent was required, in accordance with 45 CFR §46. This study follows the Strengthening the Reporting of Observational Studies in Epidemiology (STROBE) reporting guideline for cohort studies.^7^

### Study Cohort

The study cohort consisted of all adult patients (aged ≥18 years) beginning long-term dialysis in the US from 2013 to 2017 with follow-up through the end of 2018, identified in the USRDS.^8^ This study period was chosen because it reflects the recently available data from USRDS with DFC star ratings. We excluded patients with a prior kidney transplant and then we matched DFC star ratings to each annual cohort of recipients (ie, those initiating dialysis in 2014 had facility ratings that matched the 2014 report, which reports on patient data from 2012). Patients at facilities without a star rating in that year were also excluded (30 874 patients over 5 individual years of study).

### Variables

Our primary exposure variable of interest was the DFC star rating matching the year of dialysis initiation. Dialysis facility characteristics and star ratings were obtained from Medicare DFC,^9^ which contains facility characteristics, clinical outcomes related to dialysis adequacy and hospitalizations, and results from patient surveys and DFC star ratings (range, 1-5 stars). The DFC star ratings used for the present study included these quality measures^9^: standardized transfusion ratio; standardized mortality ratio; standardized hospitalization ratio; a summary measure of dialysis adequacy for adult patients undergoing hemodialysis, pediatric patients undergoing hemodialysis, and adult patients undergoing peritoneal dialysis (ie, total ratio of urea clearance multiplied by dialysis time to volume of water measure, or Kt/V); 2 measures of vascular access (fistula vs catheter); and the proportion of patients with hypercalcemia. There is no prespecified distribution of 5-star ratings for DFC with the 2016 method.

The primary outcome was the proportion of patients undergoing incident dialysis who were waitlisted within 1 year of dialysis initiation. This was specified in line with existing federal policy priorities, current quality measures (eg, the PPPW during a 1-year period), and the mandatory ETC payment model. Covariates included in the analyses were chosen for their clinical and policy importance. Patient-level covariates included sex, age, race/ethnicity, primary cause of kidney disease, body mass index, employment status, medical comorbidities, year of dialysis initiation, initial dialysis modality, and whether patients were informed of transplantation at dialysis initiation. Facility-level covariates included the number of dialysis stations, number of patients at the end of the year, for-profit status and chain ownership, and rurality. Large chain ownership was defined as being from 1 of the 3 largest dialysis chains: Fresenius and DaVita. PPPW was obtained for sensitivity analyses from the 2020 reports, the only year available.

### Statistical Analysis

We first assessed the facility-level and patient-level characteristics associated with DFC star ratings. Two-sided Pearson χ^2^ tests and Kruskal-Wallis tests were performed to determine the unadjusted associations for categorical and continuous variables. For these comparisons, patient-level characteristics were stratified by facility star rating for each year, and facility-level characteristics were individual data points at each star level in 2017 for presentation. We then mapped the variation in star ratings by urban and rural status. Counties were categorized as rural, micropolitan, or urban according to their Urban Influence Code. Next, we assessed the variation in 1-year transplant waitlisting by dialysis facility for a representative year 2017. In line with our main outcome variable, 1-year waitlisting rates were calculated on the basis of a denominator of all patients beginning dialysis for the first time (no prior transplants or dialysis) and a numerator of individuals waitlisted for kidney transplant within 1 year of dialysis start.

For our main analysis, we first assessed the unadjusted association between dialysis center quality and 1-year waitlisting. We next developed a hierarchical multivariable logistic regression model with a random effect for dialysis facility. The unit of analysis was the individual. We included patient-level covariates for risk-adjustment and facility-level factors, as described already, and controlled for secular trends with calendar year indicator. As a sensitivity analysis, we further restricted the cohort to patients more likely to not have medical, social, or dialysis facility–level contraindications to transplantation by removing patients who were (1) attending a dialysis facility with fewer than 11 patients, (2) aged 75 years or older, or (3) were admitted to nursing home currently or in the past 10 years.

An α level of .05 was used as criterion for statistical significance, and complete case analysis was used for the multivariate analyses. All statistical analyses and data linkages were performed using SAS statistical software version 9.3 (SAS Institute), and figures were produced with R statistical software version 4.0.4 (R Project for Statistical Computing). Maps were made with QGIS software version 3.12 (QGIS Development Team, Open Source Geospatial Foundation Project). Data analysis was performed from January to April 2021.

## Results

### Facility and Patient Characteristics

Over the 5-year study period (2013-2017), there were 6661 total facilities with star ratings in DFC in at least 1 study year (Table 1). A total of 4774 (71.7%) of these facilities had ratings for all 5 years of the study and were included for each individual year with ratings. The proportion of facilities in each star category ranged from 2.2% to 34.9%. Of 5869 dialysis facilities in 2017, 132 (2.2%) were 1-star, 436 (7.4%) were 2-star, 2047 (34.9%) were 3-star, 1660 (28.3%) were 4-star, and 1594 (27.2%) were 5-star (Table 1). Both medium (11-25 dialysis stations) and large (>25 dialysis stations) facilities were more commonly 4-star and 5-star facilities (χ^2^_8_ = 168.84; *P* < .001); there was no difference in the proportion of for-profit facilities across star ratings (range, 89.3%-93.2%; χ^2^_4_ = 4.35; *P* = .36). Large chain facilities tended to be 4-star and 5-star facilities (χ^2^_8_ = 244.74; *P* < .001), as was the relative proportion of rural dialysis facilities (χ^2^_8_ = 140.20; *P* < .001).

**Table 1.** Key Dialysis Facility (2017) and Patient Characteristics (2013-2017)

Covariate	Facilities or patients, No. (%)					*P* value
	1 Star (lower quality)	2 Stars	3 Stars	4 Stars	5 Stars (higher quality)	
Dialysis facility characteristics 2017, total^a^	132 (2.2)	436 (7.4)	2047 (34.9)	1660 (28.3)	1594 (27.2)	NA
Facility size						
Small (≤10 stations)	44 (33.3)	85 (19.5)	187 (9.1)	150 (9.0)	216 (13.6)	<.001^b^
Medium (11-25 stations)	82 (62.1)	310 (71.1)	1531 (74.8)	1250 (75.3)	1244 (78.0)	
Large (>25 stations)	6 (4.5)	41 (9.4)	329 (16.1)	260 (15.7)	134 (8.4)	
Total patients at end of year, median (IQR), No.	44.0 (25.2-70)	56.6 (34.0-87.0)	72.0 (45.0-105.0)	73.0 (48.0-108.0)	57.0 (37.0-90.0)	<.001^c^
Organization type						
For-profit	123 (93.2)	395 (90.6)	1830 (89.4)	1507 (90.8)	1424 (89.3)	.36^b^
Nonprofit	9 (6.8)	41 (9.4)	217 (10.6)	153 (9.2)	170 (10.7)	
Dialysis chain						
Large chain	53 (40.2)	291 (66.7)	1542 (75.3)	1333 (80.3)	1257 (78.9)	<.001^c^
Small or regional chain	11 (8.3)	43 (9.9)	245 (12.0)	162 (9.8)	169 (10.6)	
Independent	68 (51.5)	102 (23.4)	260 (12.7)	165 (9.9)	168 (10.5)	
Geographical location						
Urban	124 (93.9)	387 (88.8)	1748 (85.4)	1382 (83.3)	1163 (73.0)	<.001^b^
Micropolitan	3 (2.3)	28 (6.4)	199 (9.7)	182 (11.0)	250 (15.7)	
Rural	5 (3.8)	21 (4.8)	100 (4.9)	96 (5.8)	181 (11.4)	
Patient-to-nurse ratio, median (IQR)	11.6 (5.7-17.4)	14.5 (10.0-19.7)	15.5 (11.6-20.4)	15.2 (11.3-20.3)	14.5 (10.7-18.5)	<.001^c^
Patient-to-social worker ratio, median (IQR)	33.0 (16.5-64.0)	51.0 (27.0-78.0)	66.0 (42.0-89.6)	68.0 (44.0-91.0)	54.0 (33.0-77.0)	<.001^c^
Patients waitlisted for kidney transplant, median (IQR), %^d^						
1 y	0.0 (0.0-8.1)	0.0 (0.0-7.7)	2.9 (0.0-8.6)	3.7 (0.0-9.1)	0.0 (0.0-9.5)	.03^c^
2 y	0.0 (0.0-8.3)	3.1 (0.0-9.6)	4.3 (0.0-10.0)	4.9 (0.0-10.4)	3.6 (0.0-10.8)	.004^c^
Patient characteristics 2013-2017, total^e^	39 928 (7.9)	84 907 (16.7)	203 000 (40.0)	115 016 (22.7)	64 730 (12.8)	NA
Sex						
Male	22 437 (56.2)	48 238 (56.8)	116 682 (57.5)	66 528 (57.8)	37 917 (58.6)	<.001^b^
Female	17 491 (43.8)	36 669 (43.2)	86 318 (42.5)	48 488 (42.2)	26 813 (41.4)	
Age, median (IQR), y	66 (56-75)	65 (54-74)	65 (54-74)	65 (55-75)	66 (55-75)	<.001^c^
Race/ethnicity						
Black	12 727 (31.9)	25 339 (29.8)	57 394 (28.3)	27 264 (23.7)	11 101 (17.1)	<.001^b^
Hispanic	6753 (16.9)	11 960 (14.1)	29 553 (14.6)	17 813 (15.5)	10 913 (16.9)	
Missing	6 (0.0)	20 (0.0)	20 (0.0)	12 (0.0)	3 (0.0)	
Other^f^	1194 (3.0)	3242 (3.8)	10 717 (5.3)	8854 (7.7)	6179 (9.5)	
White	19 248 (48.2)	44 346 (52.2)	105 316 (51.9)	61 073 (53.1)	36 534 (56.4)	
Employment status						
Employed	2978 (7.5)	7832 (9.2)	19 930 (9.8)	12 065 (10.5)	6595 (10.2)	<.001^b^
Retired	24 902 (62.4)	50 902 (60.0)	122 723 (60.5)	70 294 (61.1)	39 911 (61.7)	
Unemployed	12 048 (30.2)	26 173 (30.8)	60 347 (29.7)	32 657 (28.4)	18 224 (28.2)	
Informed of transplantation at dialysis initiation	33 031 (82.7)	72 717 (85.6)	175 151 (86.3)	99 474 (86.5)	56 136 (86.7)	<.001^b^
Initial modality type						
Home hemodialysis	345 (0.9)	302 (0.4)	420 (0.2)	177 (0.2)	78 (0.1)	<.001^b^
Peritoneal dialysis	3300 (8.3)	7227 (8.5)	15 133 (7.5)	8458 (7.4)	4003 (6.2)	
In center	36 282 (90.9)	77 371 (91.1)	187 438 (92.3)	106 374 (92.5)	60 644 (93.7)	

Abbreviations: IQR, interquartile range; NA, not applicable.

^a^ Facility-level covariates are aggregated among facilities with that star rating.

^b^ Calculated with the χ^2^ test.

^c^ Calculated with the Kruskal-Wallis test.

^d^ Waitlisting was calculated as proportion of patients waitlisted who began dialysis in that year.

^e^ Patient-level covariates are stratified by star rating.

^f^ Other refers to American Indian or Alaska Native, Asian, Native Hawaiian or Pacific Islander, and multiracial.

**Figure 1.**
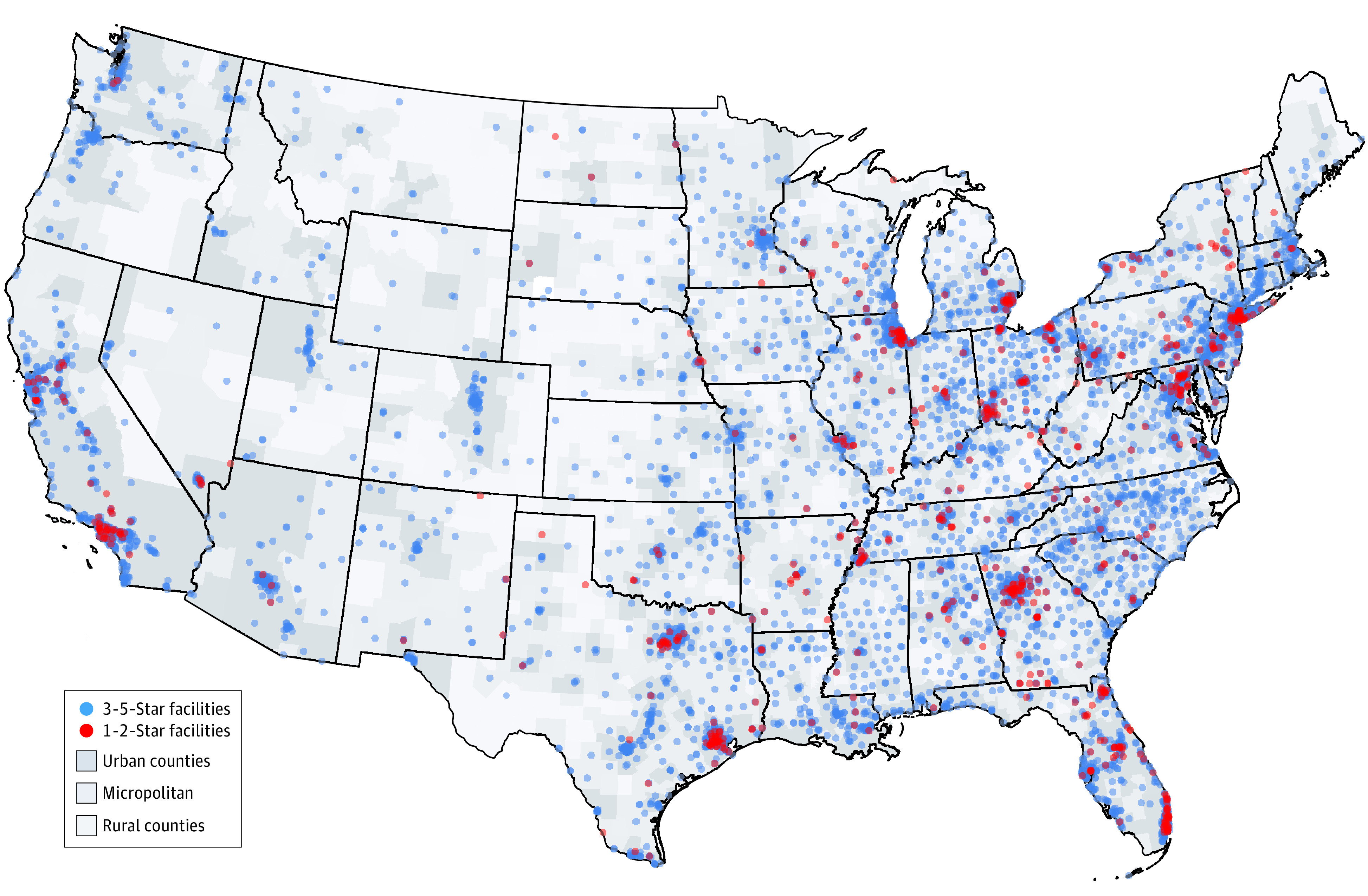
Maps of Facility Quality and Location in the US One-star and 2-star dialysis facilities (red dots) are more likely to be in urban counties (dark gray) than rural counties (light gray) (10.6% vs 6.4%). Three-star, 4-star, and 5-star facilities are denoted with blue dots. Counties were classified as rural, micropolitan, or urban according to their Urban Influence Code.

For the 507 581 patients in the study from 2013 to 2017 (291 802 men [57.4%]; 266 517 White patients [52.5%]; median [interquartile range] age, 65 [55-75] years), 180 191 patients (35.5%) received care at 4-star and 5-star facilities. The 4-star and 5-star facilities also had a lower proportion of Black patients compared with 1-star and 2-star facilities (12 727 Black patients [31.9%] attending 1-star facilities vs 11 101 Black patients [17.1%] attending 5-star facilities; χ^2^_16_ = 7606.53; *P* < .001). The 4-star (99 474 patients [85.6%]) and 5-star (56 136 patients [86.7%]) facilities also had a higher rate of informing patients of transplantation within 30 days of dialysis initiation (33 031 patients [82.7%] for 1-star facilities). The distribution of dialysis facilities by star rating varied considerably by geographical location (Figure 1). The 1-star and 2-star dialysis facilities were more likely to be in urban than rural counties (511 facilities [10.6%] vs 26 facilities [6.4%]; χ^2^_6_ = 138.17; *P* < .001) and more likely to be in major metropolitan centers vs rural areas throughout the US.

### Variation in Quality Ratings Over Time

To assess the changes in quality ratings over time, we assessed the difference between the maximum and minimum star ratings of each facility (eTable 1 in the Supplement). Of the 6661 dialysis facilities, 4251 facilities (63.8%) varied by 1 star or less. Specifically, 1462 facilities (21.9%) maintained the same star rating throughout the study, 2789 (41.9%) had a difference between the maximum and minimum rating of no more than 1 star, and 1934 (29.0%) changed by no more than 2 stars. Larger differences in maximum and minimum star ratings over time were much less common: 414 facilities (6.2%) changed 3 stars, and only 62 (0.9%) changed by 4 stars.

### Waitlisting Rates for Kidney Transplantation

**Figure 2.**
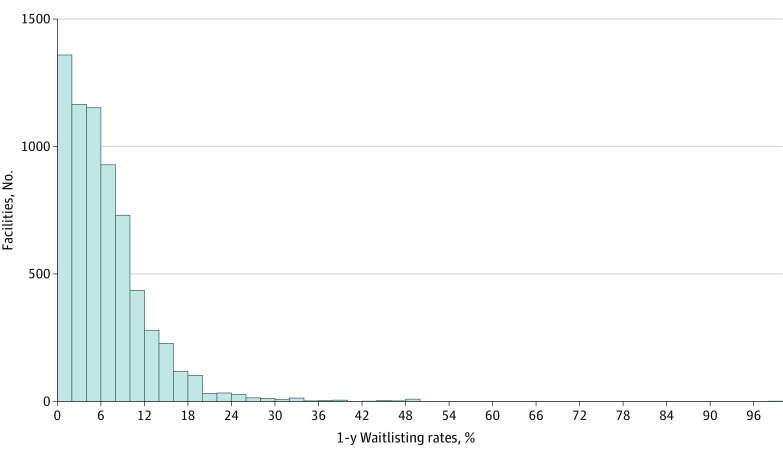
One-Year Proportion of Patients Waitlisted Among Incident Dialysis Patients by Dialysis Facility in 2017 All patients beginning dialysis for first time (no prior transplants or other dialysis) were measured as being listed for kidney transplant within 1 year of dialysis start.

The waitlisting rates for kidney transplantation start varied from 0% to 100% (median [interquartile range], 5.4% [2.5%-9.1%]) within 1 year of dialysis and from 0% to 100% (median [interquartile range], 8.7% [5.0%-13.0%]) within 2 years of dialysis (Figure 2). The median unadjusted waitlisting rate varied significantly among facilities by star rating (median, 3.6% for 5-star facilities vs 0% for 1-star facilities; χ^2^_4_ = 15.45; *P* = .004). As a sensitivity analysis, we found significant variation in the median PPPW by 2020 star ratings (median, 15.5% for 1-star facilities vs 17.6% for 5-star facilities; χ^2^_4_ = 51.62; *P* < .001) (eTable 2 in the Supplement).

The odds of waitlisting for transplantation varied considerably with dialysis facility quality rating (Table 2). There was a monotonic 47% increase in odds of waitlisting within 1 year for 5-star facilities compared with 1-star facilities (odds ratio [OR], 1.47; 95% CI, 1.39-1.57; *P* < .001). In the fully adjusted model, this corresponded to an overall difference of 2.6% vs 3.6% (difference, 1.0%; 95% CI, 0.8%-1.2%) between 1-star and 5-star facilities.

**Table 2.** Odds of Waitlisting for Kidney Transplantation Within 1 Year of Dialysis Initiation

Covariate	Unadjusted		Adjusted^a^	
	OR (95% CI)	*P* value	OR (95% CI)	*P* value
Medicare Dialysis Facility Compare rating, No. of stars				
1	1 [Reference]	NA	1 [Reference]	NA
2	1.18 (1.20-1.24)	<.001	1.12 (1.05-1.18)	<.001
3	1.28 (1.22-1.34)	<.001	1.30 (1.23-1.37)	<.001
4	1.36 (1.30-1.43)	<.001	1.42 (1.35-1.51)	<.001
5	1.34 (1.27-1.41)	<.001	1.47 (1.39-1.57)	<.001
Patient-level covariates				
Male sex	1.41 (1.38-1.45)	<.001	1.32 (1.29-1.36)	<.001
Age per year	0.94 (0.94-0.94)	<.001	0.95 (0.95-0.95)	<.001
Race/ethnicity				
Black	1.08 (1.05-1.11)	<.001	0.74 (0.72-0.76)	<.001
Hispanic	1.47 (1.43-1.52)	<.001	0.98 (0.94-1.01)	.15
Other^b^	1.63 (1.56-1.70)	<.001	1.16 (1.10-1.22)	<.001
White	1 [Reference]	NA	1 [Reference]	NA
Employment				
Unemployed	1 [Reference]	NA	1 [Reference]	NA
Employed	3.39 (3.29-3.48)	<.001	2.39 (2.32-2.47)	<.001
Retired	0.40 (0.39-0.42)	<.001	0.94 (0.91-0.97)	<.001
Informed of transplantation	3.88 (3.68-4.10)	<.001	2.28 (2.15-2.41)	<.001
Initial modality type				
In center	1 [Reference]	NA	1 [Reference]	NA
Home hemodialysis	1.61 (1.33-1.95)	<.001	1.45 (1.17-1.79)	<.001
Peritoneal dialysis	3.61 (3.50-3.71)	<.001	2.23 (2.15-2.30)	<.001
Facility-level covariates				
Facility size				
Small (≤10 stations)	1 [Reference]	NA	1 [Reference]	NA
Medium (11-25 stations)	0.98 (0.94-1.03)	.49	1.11 (1.05-1.17)	<.001
Large (>25 stations)	1.02 (0.97-1.07)	.43	1.02 (0.96-1.08)	.56
Organization type				
Nonprofit	1 [Reference]	NA	1 [Reference]	NA
For-profit	0.80 (0.77-0.82)	<.001	0.78 (0.74-0.81)	<.001
Geographical location				
Rural	1 [Reference]	NA	1 [Reference]	NA
Urban	1.82 (1.69-1.96)	<.001	1.60 (1.47-1.73)	<.001
Micropolitan	1.25 (1.15-1.36)	<.001	1.15 (1.05-1.26)	.003
Dialysis chain				
Independent	1 [Reference]	NA	1 [Reference]	NA
Large chain	0.96 (0.94-0.99)	.02	0.96 (0.92-1.00)	.03
Small or regional chain	1.09 (1.04-1.14)	<.001	1.00 (0.96-1.05)	.90
Patient-to-nurse ratio (per 10 patients)	0.96 (0.95-0.98)	<.001	0.95 (0.94-0.97)	<.001
Patient-to-social worker ratio (per 10 patients)	1.01 (1.00-1.01)	<.001	0.99 (0.98-0.99)	<.001

Abbreviations: NA, not applicable; OR, odds ratio.

^a^ Adjusted for patient and facility characteristics, other patient-level covariates, and year trend.

^b^ Other refers to American Indian or Alaska Native, Asian, Native Hawaiian or Pacific Islander, and multiracial.

In the fully adjusted model, Black patients were less likely than White patients to be waitlisted for transplantation (OR 0.74, 95% CI 0.72-0.76). Patients receiving home dialysis (OR, 1.45; 95% CI, 1.17-1.79) and peritoneal dialysis (OR, 2.23; 95% CI, 2.15-2.30) were both more likely to be waitlisted than patients receiving in-center dialysis. Patients at for-profit facilities were less likely to be waitlisted compared with patients at nonprofit facilities (OR, 0.78; 95% CI 0.74-0.81), as were patients at rural facilities compared with those at urban facilities (OR, 0.63; 95% CI, 0.58-0.68). The magnitude and direction were similar in the sensitivity analysis restricting to patients who were not in nursing homes, who were younger than 75 years, and who were at facilities with at least 12 stations (eTable 3 in the Supplement).

## Discussion

In this cohort study on the association between Medicare DFC star ratings and waitlisting of patients newly undergoing dialysis for transplant, we found that patients at 5-star rated dialysis facilities had 47% higher odds of being waitlisted compared with those at 1-star facilities. The majority of lower rated 1-star and 2-star facilities were in urban counties and served a higher percentage of patients from racial and ethnic minority groups. These findings identify areas for mitigating disparities in access to transplantation as well as improving access to transplantation via dialysis facility quality.

The findings of this study add to the literature on the importance of the dialysis facility for access to kidney transplantation, because the dialysis facility is the beginning of the referral pathway for transplantation for many patients receiving dialysis. Little literature exists on how patients are referred to particular dialysis facilities,^10^ but there is evidence that patients maintain choice of dialysis facility despite increasing overall market consolidation.^2,11^ This study adds to the considerable evidence on the role of the dialysis facility as a systems-level barrier in referral for kidney transplantation.^6,12,13,14,15,16^

Understanding the association between dialysis quality metrics and downstream process measures such as waitlisting for kidney transplantation is important with the shift toward value-based payment for patients with ESKD. Introduced as part of Advancing American Kidney Health, the ETC model adjusts Medicare dialysis payments across a random selection of 30% of Hospital Referral Regions between January 2021 and June 2027 via a performance payment adjustment influenced by the PPPW.^17,18^ We also found an association between higher PPPW and 2020 star ratings.

There is precedent for improving dialysis facility quality: Salerno et al^19^ showed that the percentage of 4-star and 5-star dialysis facilities increased substantially (from 30.0% to 53.4%) over a 3-year period as the result of improvements in dialysis adequacy and hypercalcemia measures. Our findings support the measurement of dialysis facility quality as means to accessing kidney transplantation and extend as a strategy to increase access to kidney transplantation by improving the quality of dialysis facilities.

Our finding of a disproportionate share of patients from racial and ethnic minority groups receiving care at low-quality of dialysis facilities uncovers an additional mechanism for persistent disparities in kidney transplantation. Despite decades of wide recognition, policy reforms, and extensive research, the rates of waitlisting have not meaningfully improved over 2 decades in the US,^20^ which has been consistently demonstrated in the most at-risk populations: racial and ethnic minority groups, patients with lower socioeconomic status, rural patients, older patients, and patients with comorbidities.^16,21,22,23,24^ Our findings suggest that one source of the disparity is upstream from waitlisting at the transplant center: patients from racial and ethnic minority groups are more likely to receive care at low-quality dialysis facilities, which are, in turn, associated with lower transplant waitlisting rates. The barriers to accessing transplantation and high-quality dialysis facilities are deeply embedded in the health care system.

The literature on the role of quality star ratings in improving quality of care in Medicare has been mixed. Quality star ratings in general have engendered criticism because of concerns over clinical relevance and substantial gaming of the underlying metrics.^25^ For hospitals, higher star ratings have been correlated with objective clinical outcomes such as mortality and readmissions.^26^ For skilled nursing facilities, quality star ratings have highlighted the substantial geographical variation in quality of skilled nursing facilities, as well as associations between facility quality and lower socioeconomic communities.^27,28^ However, for insurance plans, higher-rated plans have been associated with improved patient access to higher-quality hospitals.^29^ In light of the controversies on the use of quality star ratings, our findings provide reassurance to policy makers, clinicians, and patients that the Medicare DFC star ratings are associated with clinical process measures of quality along the care continuum and may be useful information for patients to select higher-quality dialysis facilities. There may also be potential for public reporting to motivate improvement in the quality of care delivered at dialysis facilities. However, there needs to be ongoing, critical evaluation of the underlying metrics to ensure that the star ratings are truly reflective of high-quality, patient-centered care.

Our findings provide important insights for federal policy makers. First, the large variation in 1-year waitlisting across dialysis facilities represents a major target for improvement because of both the average gap between current and target-levels of transplant waitlisting and the substantial performance gap between high-quality and low-quality dialysis facilities. One approach to incentivize improvement would be to include 1-year transplant waitlisting rates as a component of the DFC star ratings themselves. In addition, our findings suggest a clear link in quality of care between the care continuum of ESKD to transplantation, and efforts at quality measurement and value-based payment should straddle both the dialysis and transplantation sites of care for patients with ESKD. Third, given the association between racial and ethnic minority status and decreased likelihood of 1-year waitlisting, which appears to be mediated by access to lower-quality dialysis facilities, improving access to high-quality dialysis facilities or improving the quality of dialysis facilities used by patients from racial and ethnic minority groups may increase the equity of care for patients with ESKD. Finally, we recommend that metrics should not disproportionately penalize facilities serving patients with higher social risk.^30^

### Limitations

Our analysis is limited by its retrospective nature, which cannot establish causal relationships. On the basis of reporting requirements from DFC, there are incomplete data on small facilities. Although capture of transplant waitlisting is complete via national transplant registries, this is not the only measure of access to kidney transplantation data because transplant programs play a large role in waitlisting decisions that may be outside the control of the patient and the dialysis facility. Our models do not specifically consider local or regional policy initiatives that could affect the likelihood of waitlisting for kidney transplant. Furthermore, because USRDS data are limited to patients receiving dialysis, it does not capture access to kidney transplantation for patients with advanced kidney disease whose renal function is poor enough to qualify for transplant (eg, with an estimated glomerular filtration rate < 20 mL/min/1.73 m^2^) but not receiving long-term dialysis.

## Conclusions

We provide evidence that Medicare DFC ratings capture an important dimension of quality. Our study suggests that policy makers should consider quality metrics that reflect not just the site of care delivery itself but also incentivize coordination of care along the patient care continuum. Moreover, the wide variability in the proportion of waitlisted patients suggests that this may be an important target for a quality measure. In the context of changing payment models that are intended to improve ESKD care, attention to the association between dialysis facility quality and transplantation should guide future policy.

## Notes

Supplement. **eTable 1.** Minimum and Maximum 5 Star Ratings Between 2013-2017 **eTable 2.** Comparing Median 2020 Percentage of Prevalent Patients Waitlisted (PPPW) by 5 Star Ratings **eTable 3.** Sensitivity Analysis for Odds of Waitlisting for Kidney Transplantation Within One Year of Dialysis Initiation (Adjusted for Other Patient-Level Covariates, as Well as for Year Trend)
