# Supplementary material for: Association of Public Reporting of Medicare Dialysis Facility Quality Ratings With Access to Kidney Transplantation
Source: JAMA Netw Open. 2021 Sep 24;4(9):e2126719. doi: 10.1001/jamanetworkopen.2021.26719 (PMC8463939; doi:10.1001/jamanetworkopen.2021.26719)
Supplement: Supplement. — eTable 1. Minimum and Maximum 5 Star Ratings Between 2013-2017 eTable 2. Comparing Median 2020 Percentage of Prevalent Patients Waitlisted (PPPW) by 5 Star Ratings eTable 3. Sensitivity Analysis for Odds of Waitlisting for Kidney Transplantation Within One Year of Dialysis Initiation (Adjusted for Other Patient-Level Covariates, as Well as for Year Trend) [file jamanetwopen-e2126719-s001.pdf]

Supplemental Online Content

Adler JT, Xiang L, Weissman JS, et al. Association of public reporting of Medicare dialysis facility quality ratings with access to kidney transplantation. *JAMA Netw Open*. 2021;4(9):e2126719. doi:10.1001/jamanetworkopen.2021.26719

eTable 1. Minimum and Maximum 5 Star Ratings Between 2013-2017

eTable 2. Comparing Median 2020 Percentage of Prevalent Patients Waitlisted (PPPW) by 5 Star Ratings

eTable 3. Sensitivity Analysis for Odds of Waitlisting for Kidney Transplantation Within One Year of Dialysis Initiation (Adjusted for Other Patient-Level Covariates, as Well as for Year Trend)

This supplemental material has been provided by the authors to give readers additional information about their work.

© 2021 Adler JT et al. *JAMA Network Open.*

eTable 1: Minimum and Maximum 5 Star Ratings Between 2013-2017 [a, b]

		Maximum				
		1 star	2 stars	3 stars	4 stars	5 stars
Minimum	1 star	148(2.2)	211(3.2)	397(6.0)	141(2.1)	62(0.9)
	2 stars		186(2.8)	829(12.5)	503(7.6)	273(4.1)
	3 stars			583(8.8)	1150(17.3)	1034(15.5)
	4 stars				240(3.6)	599(9.0)
	5 stars					305(4.6)

[a] Maximum and minimum Dialysis Facility Compare star ratings for the facilities included in the study. [b] All values expressed as (n, %) of facilities with those ratings during the study.

eTable 2: Comparing Median 2020 Percentage of Prevalent Patients Waitlisted (PPPW) by 5 Star Ratings

	Percentage of Prevalent Patients Waitlisted (PPPW)	
**Dialysis Facility Quality Star Rating**	**Median**	**IQR**
1	15.5%	(7.60-23.60)
2	14.8%	(8.95-22.55)
3	15.7%	(10.50-23.00)
4	17.3%	(11.60-24.70)
5	17.6%	(11.65-25.20)

Kruskal-Wallis tests for medians: **p-value<0.001**

© 2021 Adler JT et al. *JAMA Network Open.*

eTable 3: Sensitivity Analysis for Odds of Waitlisting for Kidney Transplantation Within One Year of Dialysis Initiation (Adjusted for Other Patient-Level Covariates, as Well as for Year Trend).

	**Adjusted for patient and facility characteristics**	
**Covariate (reference)**	**Odds ratio (95% CI)**	**P value**
DFC star rating (1 star)		
2 stars	1.12 (1.05-1.18)	<0.001
3 stars	1.30 (1.23-1.37)	<0.001
4 stars	1.42 (1.34-1.50)	<0.001
5 stars	1.47 (1.38-1.56)	<0.001
Male sex	1.30 (1.27-1.34)	<0.001
Age (per year)	0.96 (0.96-0.96)	<0.001
Race (white)		
Black	0.73 (0.71-0.75)	<0.001
Hispanic	0.97 (0.94-1.00)	<0.09
Other	1.16 (1.10-1.22)	<0.001
Employment (unemployed)		
Employed	2.32 (2.25-2.40)	<0.001
Retired	0.96 (0.93-0.99)	0.010
Informed of transplantation	2.10 (1.99-2.23)	0.01
Initial modality type (in-center)		
Home hemodialysis	1.46 (1.18-1.80)	<0.001
Peritoneal dialysis	2.19 (2.12-2.27)	<0.001
For-profit (non-profit)	0.78 (0.75-0.82)	<0.001
Geography (rural)		
Urban	1.61 (1.49-1.75)	<0.001
Micropolitan	1.16 (1.05-1.27)	0.002
Dialysis chain (independent)		
Large chain	0.96 (0.92-1.00)	0.04
Small/regional chain	1.00 (0.95-1.05)	0.98
Patient: nurse ratio (per 10 patients)	0.95 (0.93-0.96)	<0.001
Patient: social worker ratio (per 10 patients)	0.99 (0.98-0.99)	<0.001

This matches same population as Table 2, but has the following additional exclusions:
- Patients in dialysis facility with less than 11 patients
- Patients with age ≥ 75
- Patients who admitted to nursing home currently or in the past 10 years

© 2021 Adler JT et al. *JAMA Network Open.*
